# Circadian Plasticity in Photoreceptor Cells Controls Visual Coding Efficiency in *Drosophila melanogaster*

**DOI:** 10.1371/journal.pone.0009217

**Published:** 2010-02-15

**Authors:** Martin Barth, Michael Schultze, Christoph M. Schuster, Roland Strauss

**Affiliations:** Friedrich-Miescher-Laboratory of the Max-Planck Society (MPG), Tuebingen, Germany; Pennsylvania State University, United States of America

## Abstract

In the fly *Drosophila melanogaster*, neuronal plasticity of synaptic terminals in the first optic neuropil, or lamina, depends on early visual experience within a critical period after eclosion [1]. The current study revealed two additional and parallel mechanisms involved in this type of synaptic terminal plasticity. First, an endogenous circadian rhythm causes daily oscillations in the volume of photoreceptor cell terminals. Second, daily visual experience precisely modulates the circadian time course and amplitude of the volume oscillations that the photoreceptor-cell terminals undergo. Both mechanisms are separable in their molecular basis. We suggest that the described neuronal plasticity in *Drosophila* ensures continuous optimal performance of the visual system over the course of a 24 h-day. Moreover, the sensory system of *Drosophila* cannot only account for predictable, but also for acute, environmental changes. The volumetric changes in the synaptic terminals of photoreceptor cells are accompanied by circadian and light-induced changes of presynaptic ribbons as well as extensions of epithelial glial cells into the photoreceptor terminals, suggesting that the architecture of the lamina is altered by both visual exposure and the circadian clock. Clock-mutant analysis and the rescue of PER protein rhythmicity exclusively in all R1-6 cells revealed that photoreceptor-cell plasticity is autonomous and sufficient to control visual behavior. The strength of a visually guided behavior, the optomotor turning response, co-varies with synaptic-terminal volume oscillations of photoreceptor cells when elicited at low light levels. Our results show that behaviorally relevant adaptive processing of visual information is performed, in part, at the level of visual input level.

## Introduction

The light response of photoreceptor cells and/or the neuronal computation of their output functions are often modulated so as to optimize vision under varied and changing light conditions. Underlying mechanisms range from structural changes of the light-sensitive cells over differences in conductance of photoreceptor membranes, or in synaptic signal transduction, up to altered levels of post- to presynaptic feedback modulation. One well described example for the first mechanism is provided by the horseshoe crab, *Limulus polyphemus*, in which the sensitivity of photoreceptor cells in the lateral eye is controlled by structural and physiological changes in the receptors [2]. Turnover of photoreceptor membranes, shape of quantum bumps, or temporal response properties are among the well-orchestrated mechanisms that serve to increase the chance of an incident photon to strike a rhodopsin molecule. These mechanisms increease signal to noise ratio and thereby visual sensitivity.

Neuronal computational mechanisms, like those in the vertebrate retina [3], enable animals to optimize visual behavior. For instance, compensation for the delayed visual response of the eye to the trajectory of a moving object occurs in the retina rather in the visual cortex. The neuronal flexibility of retinal ganglion cells ensures that the retina encodes the invariant features of objects regardless of changing ambient lighting, and thus provides the organism continuously with optimal visual information.

**Figure 1:**
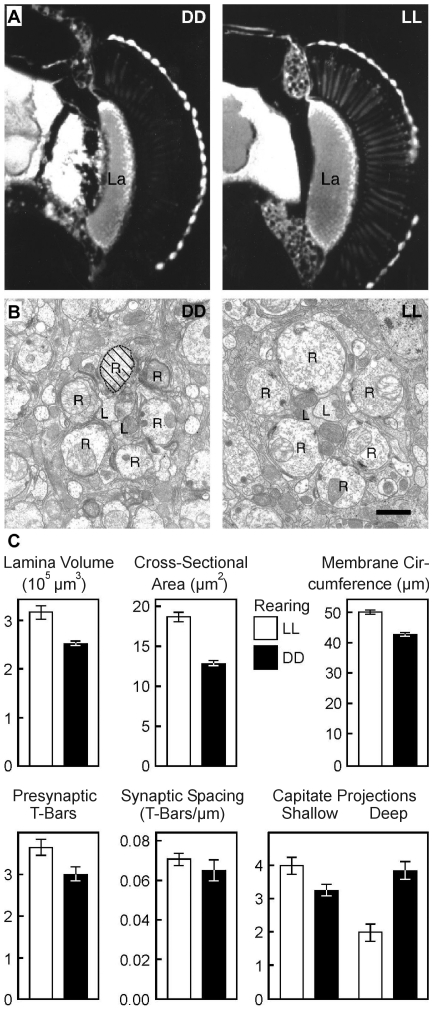
Experience-dependent plasticity in the lamina of wild-type *Drosophila melanogaster*. **a**, Autofluorescence profiles of the lamina neuropil (La) from adult flies reared for four days in constant darkness (DD) or constant light (LL; flies were fixed at ZT 6). Note the gross morphological size changes of the laminae. **b**, Electron-micrographs of distal lamina sections of flies sectioned at day and night. Six synaptic terminals of photoreceptor cells (R) converge on to two Large Monopolar Cells (L), forming the so called cartridge. Scale bar, 1 µm. **c**, Significant differences between LL- (white columns) and DD-flies (black columns) were found for lamina volume, cross-sectional area of the photoreceptor terminals (1/6 hatched in b), circumference of the membrane surrounding these terminals (1/6 dotted line in b) and presynaptic T-bars residing on this membrane. Spacing between neighboring synapses was statistically indistinguishable. Finally, in LL-flies more shallow capitate projections were found, whereas in DD-flies more deep capitate projections invaded the photoreceptor terminals. Number of presynaptic T-bars and capitate projections were counted per section. Error bars denote s.e.m. values.

In the fruit fly, *Drosophila melanogaster,* and in other insect species, the first optic neuropil, or lamina, is a potential site for such adaptive neuronal coding. It was reported that visual stimulation early in adult life increases the size of both optic lobes in *Drosophila melanogaster* [1] and certain brain regions [4], suggesting that visual experience during a critical period is involved in fine-tuning the development of neuronal circuitry. Specifically, the lamina is largest in flies reared in constant light (LL) and smallest in those reared in constant darkness (DD; see also Fig. 1a). These gross morphological changes are accompanied by corresponding volume differences in photoreceptor cell terminals [3]. In addition to this earlier investigation, we now studied also the frequency of presynaptic ribbons [5] and extensions of epithelia glial cells [deep and shallow capitate projections; 6], [7] into or onto the photoreceptor cell terminals. Altogether, the results of this investigation lead to the assumption that not only is the volume of photoreceptor cell terminals highly modifiable, but so is the whole neuronal architecture of the lamina of *Drosophila melanogaster*.

Early visual experience has been shown to be of major importance for the development of the visual system in *Drosophila melanogaster*, larger flies and other insect species [8]. Yet, different light levels at day and night pose another challenge for most animals. Considering the neuronal plasticity we have described before, *Drosophila* with its major activity peaks during the daylight-transition phases at dawn and dusk provides a valuable model system to study the circadian neuronal mechanisms of visual system- and visual behavior adaptation. In *Drosophila*, there is already evidence for rhythmic size changes in the first optic neuropil where the axons of the large monopolar cells L1 and L2 swell at the beginning of both day and night and shrink during the courses of day and night, respectively [9]. Moreover, L2 shows daily changes in the morphology of its dendritic spines, and are most pronounced at the beginning of the night [10]. Recently, circadian remodeling in the axonal terminals of the PDF circuit has been shown, with higher complexity during the daytime [11].

Further support comes from the housefly *Musca domestica* where there is evidence for circadian structural changes in its visual system [12]. The impact of the circadian changes on the behavioral output of these animals remained, however, unclear.

In this paper we studied the response of the first optic neuropil to circadian variations in light levels and then tested their behavioral relevance. We used a simple, well-described visually guided behavior in *Drosophila melanogaster* by recording the rudder-like deflections of their abdomen in response to visual stimulation [13] at varying light intensities. By studying *Drosophila* clock mutants and a mutant defective in the phototransduction cascade, we unraveled some of the underlying cellular and molecular mechanisms, enabling us to describe a system of circadian and developmental neuronal plasticity together with its behavioral implications.

## Materials and Methods

### Fly Stocks and Rearing

The following fly strains were raised on cornmeal medium at 25°C: wild-type Canton-S (WT CS), *period* (*per^01^*), *timeless* (*tim^01^*), *no receptor potential A* (*norpA^P24^*) and *per^01^; Rh1(-180)-per−1/+*. The latter were crossed into a *per^01^* background.

**Figure 2:**
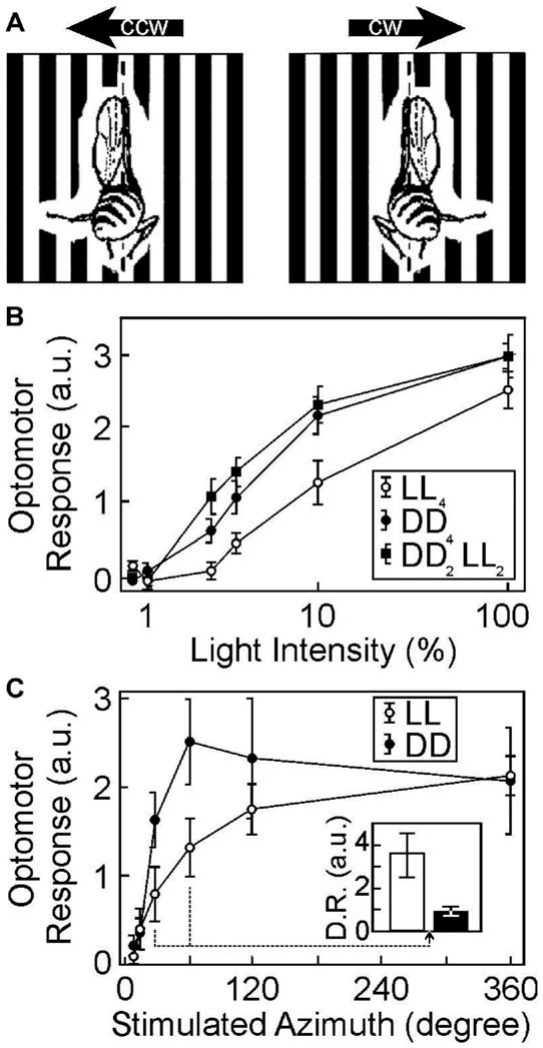
Sensitivity and dynamic range of optomotor behavior depends on early visual experience. **a**, The optomotor response: A tethered female fly attempts to follow the rotation of a periodical pattern of 27.7° width at 2.6 Hz contrast frequency and thereby deflects its abdomen. Flies were stimulated by both counter-clockwise (CCW) and clockwise (CW) rotation of the cylinder and the behavioral net response was calculated [13]. **b**, Optomotor responses were maximal at about 10 cd m^−2^ ( = 100%) for all tested fly groups. At low light intensities behavioral responses of flies reared in DD_4_- or DD_2_LL_2_-conditions (two days darkness followed by two days light) were statistically indistinguishable, with both displaying a higher optomotor sensitivity than LL_4_-reared flies. Flies were kept under LL-conditions before eclosion. **c**, The stimulus was restricted in the azimuth direction to various angular ranges of the flies' frontal visual field. With increasing area of frontal stimulation the optomotor responses of LL-reared flies increased continuously in a fine-graded manner, whereas the optomotor response of DD-reared flies saturated already at 45° of stimulated azimuth (n = 8 to 11 flies per condition). The dynamic range (D.R.), computed as the quotient of the optomotor response at 360° stimulation versus that at 30°–60°, was therefore higher in LL-flies than in DD-flies.

In experiments designed to study changes in lamina volume, flies were kept in 12:12 h light-dark (LD)-conditions as larvae and pupae and, upon eclosion, were kept for 4–6 days under one of the following conditions: constant light (LL), cycling light/dark (LD), constant darkness (DD). In the experiment designed to study the behavioral effects of constant light and darkness in adulthood (e.g. Fig. 2), flies were kept in constant light throughout larval and pupal stages and, upon eclosion, were kept for 4 days under one of the following conditions: LL, DD or DD_2_LL_2_ (two days darkness followed by two days light). Illumination by full-spectrum fluorescent light was provided with an average intensity of about 500 cd/m^2^ flickering at 20 kHz.

### Quantification of Optomotor Turning Responses

Optomotor turning responses were measured in tethered, stationary flying animals by recording the rudder-like deflections of the abdomen. Graded bending of the abdomen toward the intended side is one component of the natural flight steering behavior [13], [14]. It is consistently found under open-loop conditions in which the steering maneuvers of the fly do not alter its visual stimulation. To achieve stationary flight, test flies were mounted to a piece of 0.2 mm diameter tungsten wire under cold anesthesia (+4°C) in a stream of dry air. A droplet of light-sensitive glass glue (Loctite) on the tip of the wire was applied from above in the gap between the fly's head and thorax and cured with 30 s of UV irradiation.

The tethered fly was suspended in the center of a cylindrical projection screen. The abdomen was illuminated with red light (which is invisible to *Drosophila*) and viewed from above through a black-and-white CCD-camera with macro optics and a red filter to suppress background illumination. Two photovoltaic cells each with a rectangular aperture were attached to the screen so that the long axis of their active areas coincided with the lateral edges of the abdomen. When the fly deflected its abdomen towards one side while turning, the bright image of the abdomen against a dark background increased the illumination of the photovoltaic cell on the inner side of the intended turn and at the same time the dark background decreased the illumination of the cell on the other side. A differential amplifier generated a combined electrical signal from the two cells that corresponded in magnitude and sign to the fly's turning. Details of the apparatus are described elsewhere [13]. A grid consisting of about 27 seven degree wide, equally spaced, black bars on a light background was projected from below onto the cylindrical screen and rotated with constant speed (contrast frequency: 2.6 Hz) around the fly. Flies were stimulated with both counter-clockwise (CCW) and clockwise (CW) rotation of the stripes and the net turning response was computed.

### Tissue Sectioning and Lamina Volumetry

Circadian changes in lamina volume were measured using techniques described previously [1]. Briefly, 10–20 flies were sacrificed and prepared for histology at three hour intervals throughout a 24-hour period. After fixation, serial frontal cross-sections of lamina were visualized with fluorescence microscopy and measured the full length of the structure. (e.g. Fig. 1a) Area measurements and volume reconstructions were performed by an experimentally blind observer using a custom computer program kindly provided by R. Wolf and M. Heisenberg, Univ. Wuerzburg [15]. Statistical analysis see below.

### Electron Microscopy

At the appropriate age and time, flies were etherized, decapitated, their proboscis removed, and the head dissected. One half of each head was processed so that only one eye per fly was sectioned and analyzed. Tissue was fixed in a cacodylate-buffered glutaraldehyde-paraformaldehyde primary fixative, osmicated and embedded in Epon. Embedded eyes were oriented to allow tangential sectioning of the lamina. In an attempt to collect only sections from the distal side of the lamina, semithin sections were cut until the first cartridges within the lamina neuropil could be seen after which ultrathin 65 nm sections were made.

### Quantification of Histological Sections

**Figure 3:**
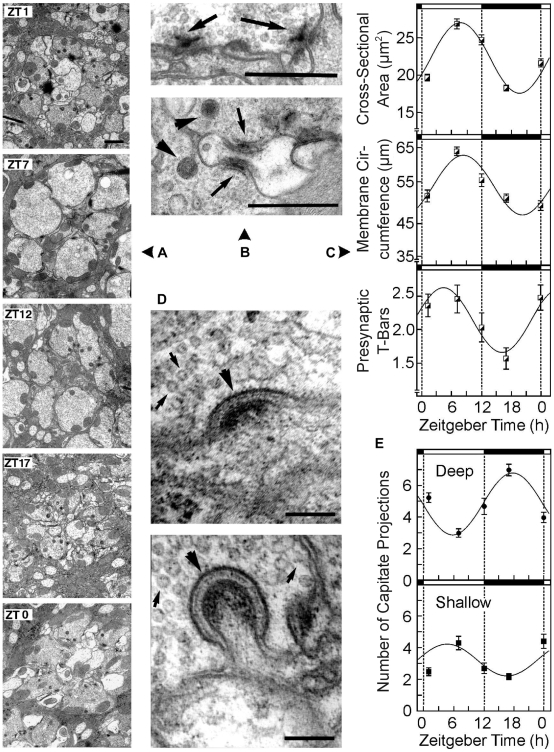
Day/night structural changes of photoreceptor cells and capitate projections. **a**, Typical cross-sectional profiles of cartridges at the distal lamina. At ZT 7 the axonal profiles reach their largest cross-sectional area, in the middle of the night (ZT 17) they are smallest. Scale bar, 1.0 µm. **b**, Examples of presynaptic ribbons of tetrad synapses (arrows; arrowheads, capitate projections). Scale bar, 0.5 µm. **c**, The analysis of photoreceptor cell area, membrane circumference and residing presynaptic T-bars shows robust changes during the 24-hour cycle (mean size ± s.e.m. of 40–55 photoreceptor cells; about 10 cartridges per fly; n_ZT1_ = 5, n_ZT7_ = 5, n_ZT12_ = 5, n_ZT17_ = 6, n_ZT0_ = 5 flies were analyzed for each point of time). **d**, Capitate projections of epithelial glial cells (arrowheads) were distinguished based on their position relative to the photoreceptor terminal. Shallow capitate projections (above) embrace the axon on its surface, deep capitate projections (below) extrude deep into the photoreceptor cell terminal. Small arrows indicate synaptic vesicles inside the photoreceptor cell axon. Scale bar, 0.1 µm. **e**, Shallow capitate projections were found to be most abundant during day times, deep ones during night times. Fitting the data to 24-hour sine-waves revealed that the two curves were in anti-phase by about 12 h.

Cartridges were viewed in the electron microscope and single cartridges were photographed at 11.500× magnification on 80-mm negative film. Negatives were scanned (Snapscan 600, Agfa) into Adobe Photoshop and then processed for final morphometric analysis in IpLab. Processing was done blindly so that the observer did not know the rearing history of the fly at the time when lamina sections were measured. Within each cartridge cross-section we measured the membrane circumference and surface area of each photoreceptor cell terminal (R1-6) and counted the presynaptic ribbons residing on the membrane. In an initial experiment, we analyzed only those presynaptic ribbons [5] that were sectioned in an exact transverse plane and showed pedestral and platform (Fig. 3b). The sections were then analyzed a second time and synapses that were obliquely sectioned were counted. There was a strong positive correlation between the counts obtained using the two criteria. In subsequent experiments, we therefore did not differentiate between oblique and transverse sections. For each cartridge, we determined the number of synapses contained in a single section. For each cartridge, the ratio between the number of synapses and the circumference of the photoreceptor membrane was used to determine the average spacing between synapses. This method provides an indirect comparison of different test groups. In order to quantify the number of synapses and their spacing on a two-dimensional membrane surface, one would have to do serial reconstructions of cartridge sections [16]. Finally, we counted the invaginations of epithelial glia cells, deep and shallow capitate projections [6], [7], into or onto the photoreceptor cell terminals (Fig. 3d). Because the section thickness influences the number of visible synapses and capitate projections, we took great care to keep this parameter as uniform as possible. Differences in absolute numbers between comparable groups in different experiments (compare e.g. Fig. 1 and 3) might derive from small variations in the histological treatment between different experiments. **Statistical analyses**. For each experimental group at least five flies were sectioned (one eye of each fly) and analyzed quantitatively. Since variations between flies appeared to be larger than within a given animal, we analyzed on average 5–10 cartridges per fly. Samples of one experimental group therefore contained on average data from up to 50 cartridges, i.e. 300 photoreceptor cell terminals. Statistics were performed using the STATISTICA program.

### Statistical Tests for Circadian Patterns

**Figure 4:**
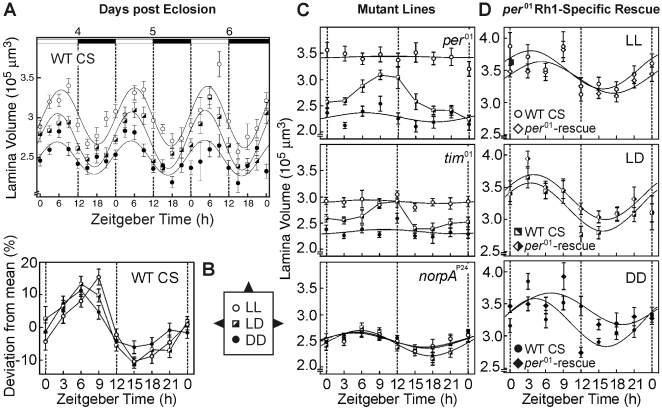
Structural plasticity of the lamina is controlled by the parallel action of a circadian clock and a phototransduction-dependent mechanism. **a**, Lamina volumes of adult wild-type CS (WT CS) flies kept under LL, LD, or DD conditions during adult life. All flies had experienced 12:12 LD cycles throughout larval and pupal development. The lamina volume oscillated in a circadian manner around a mean value which was larger in light-experienced flies than in dark-reared flies (F(2,1699)  = 156.7, p<0.0001, ANOVA). Sinusoidal-function fitting revealed a best fitting period of 23.7 h for LL (F(1,563) = 17.5, p<0.0001) and DD (F(1,515) = 5.6, p<0.05) lamina volume oscillations and 24.0 h for LD (F(1,621) = 34.2, p<0.0001). ‘Circadian Time’ (for LL and DD) ZT 0  =  light on, ZT 12  =  light off; for LL- and DD-flies read ZT as ‘Circadian Time’. **b**, Calculated amplitude deviations from the respective mean lamina volumes of all data points in a. Light experience significantly modified the time course of the circadian lamina-volume oscillations. This is most apparent at ZT 9, where LL reared flies showed the highest volume and DD reared flies the lowest volume. **c**, Lamina volumes of 10 to 15 adult mutant male flies per point of time kept under LL, LD or DD conditions. Circadian volume oscillations of the lamina were absent in the two clock mutants *per^01^* and *tim^01^* reared under LL- or DD-conditions. LD-reared animals displayed an increase in lamina volume during the day and a rapid decrease at night. The blind *norpA^P24^* mutant displayed a robust circadian rhythm of lamina volumes. Under all rearing conditions the mean lamina volume rested at the DD-level of wild-type flies. **d**, Rescue of circadian lamina-volume oscillations in *per^01^*-mutants by the transgenic expression of PER-protein exclusively in photoreceptor cells R1-6 using *per^01^; Rh1(−180)-per−1/+* flies. LD-reared R1-6-rescue flies displayed lamina-volume oscillations which were statistically indistinguishable in amplitude and time course from those of WT CS flies. Constant LL - or DD-conditions delayed the subjective day peaks by several hours in R1-6-rescued animals compared to wild-type flies. These observations are consistent with a previously described delay of the PER-protein cycle under DD-conditions in animals of the same genotype. For genetic reasons, in this experiment female flies were used. They were raised under LD-conditions up to their second day of adulthood before being reared for two more days in LL, LD or DD (n = 8 to 10 per point of time). Note that the overall lamina volume in females is higher than in males.

**Figure 5:**
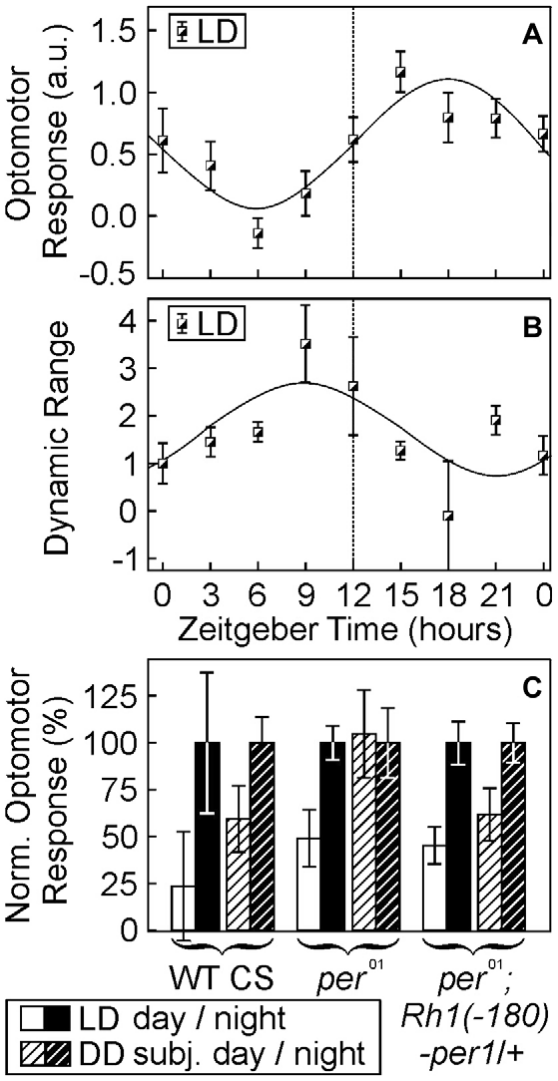
Sensitivity and dynamic range of optomotor behavior is controlled by experience-dependent and circadian mechanisms. **a, b**, LD-reared WT flies were behaviorally tested in the paradigms described in Fig. 2. Both, the sensitivity of the optomotor response at low light intensities (a, n>16 per point of time) and the dynamic range of optomotor behavior (b, n>30 per point of time) oscillated in a circadian, anti-phasic manner: highest behavioral sensitivity at night was accompanied by lowest dynamic range, and vice versa. **c**, Rescue of circadian oscillations in optomotor sensitivity by the restoration of the circadian clock in photoreceptor cells (Fig. 4d). Adult flies of the indicated genotypes were reared as described above and were behaviorally tested during their night- or day-phases (ZT 18–21 [set to 100%] and ZT 6–9, respectively). Irrespective of the genotype all LD-reared flies displayed a higher optomotor sensitivity during the night (F(1,74) = 14.6, p<0.0005). DD-reared wild-type flies as well as clock-rescued flies (*per^01^; Rh1(−180)-per−1/+*) showed a similar subjective day/night difference in behavioral sensitivity, which was absent in dark-reared *per^01^*-mutants. Error bars indicate s.e.m. values.

Volumetric and behavioral data were fitted to a sinusoidal function (custom computer program by R. Wolf and M. Reif, Univ. Wuerzburg) followed by a least-square regression analysis to estimate whether the distribution of data better fits a sinusoidal curve or a flat line through the average of the data points (paired t-test). Subsequently, the data were fitted to sine waves of varying frequencies in order to determine the frequency with the best fit. In the corresponding figures (Fig. 3, 4, 5) we only present the best fit sine wave function.

## Results

### Visual Experience-Dependent Plasticity of the Lamina and Behavioral Correlates Thereof

Volume differences in the lamina due to rearing in different light conditions have been reported before [1]; they find their counterpart in corresponding changes in cross-sectional profiles of photoreceptor cell terminals in the lamina [1]. Since these findings build a cornerstone of the present study, again, figures of lamina profiles and cross-sections from the lamina are shown in Fig. 1a and b. We have now extended this previous investigation on the effects of visual experience on photoreceptor cell terminal size at the distal lamina by measuring experience-dependent changes in synaptic frequency of tetrad synapses and glia cells at the electron microscopic level. As in this earlier study [1] the cross-sectional area of photoreceptor cell terminals was significantly larger (about 40%) in LL-flies than in DD-flies (F(1,604) = 79.8; p<0.0001; t-test). This increase of axon cross-sectional area was reflected by a 17% increase in membrane circumference (Fig. 1c). In addition, the number of synapses (presynaptic T-bars; see also Fig. 3b) residing on the membrane increases at about the same rate (22.5%), resulting in a significant difference in synaptic frequency between LL- and DD-flies (F(1,137) = 7.2; p<0.01; Fig. 1c). Synaptic spacing, calculated as the distance between two neighboring synapses on a single cross-sectional profile, did not change as a result of differential adult rearing conditions: on average, in LL-flies we encountered one synapse every 14.1 µm, and in DD-flies one every 15.4 µm (F(1,99) = 1.1; p>0.2; t-test; Fig. 1c).

We also analyzed experience-dependent changes in extensions of epithelial glial cells, so-called capitate projections. Histological descriptions of capitate projections [e.g. 6], [7] distinguish between those reaching deep into photoreceptor cells (‘deep capitate projections’; Fig. 3d below), and those embracing a photoreceptor cell on its surface (‘shallow capitate Projections’; Fig. 3d above). We found a significant interaction between the relative distribution of both types and the adult LL- and DD-rearing conditions (F(1,280) = 30.5; p<0.0001; 2-way ANOVA): Shallow capitate projections were found significantly more often in LL-flies than in DD-flies (F(1,140) = 6.4; p<0.05; Fig. 1c), whereas about twice as many deep capitate projections invaded the photoreceptor terminals in DD-flies than in LL-flies (F(1,140) = 25.8; p<0.0001; Fig. 1c).

The latter finding suggests that early visual experience not only affects the relative volume of the cells, but the whole architecture of the lamina neuropil. In dark-reared flies, small photoreceptor axons are invaginated by more projections from epithelial cells. These findings raise the possibility that differential exposure to visual stimuli modulates the interactions between neurons and glia since volume changes of photoreceptor cells are associated with changes in glial invaginations.

In order to look for behavioral correlates of these experience-dependent morphological changes we measured the optokinetic response, which involves the lobula plate [17], in flies reared in these different light regimes. This response, measured by compensatory turning of a tethered fly during movement of the visual scene (Fig. 2a), has been used as a behavioral measure of visual system function. We hypothesized that optomotor responses might provide a “readout” for experience-dependent optimization of photoreceptor terminals in the lamina. If so, we would expect flies reared in darkness to function better at low light levels than their counterparts reared in constant light.

In all groups tested we observed maximal optomotor turning responses at a stimulus intensity of 10 cd m^−2^ (Light intensity at 100% in Fig. 2b), and used this level as our baseline. Differences between the fly groups occurred at light intensities below 10 cd m^−2^. We found that animals kept in darkness during the first four days of adult life (DD), as well as those put into the light after two days in darkness (DD_2_LL_2_ in Fig. 2b) responded significantly more strongly at lower light levels (below 10 cd m^−2^) than did light-reared controls (LL; F(2,191) = 8.5, p<0.0005, ANOVA; Fig. 2b). Experience-dependent changes in lamina volume followed a similar pattern in that the lamina was smaller in both DD and DD_2_LL_2_ flies than in LL controls [1]. Thus, experience-dependent behavioral changes and lamina volume are correlated. Furthermore, the behavioral changes persist after two days in the light and thus do not reflect short term modifications such as light adaptation, light-induced reduced photoreceptor sensitivity or photoreceptor desensitization. Our findings suggest that optomotor performance reflects photoreceptor cell terminal volumes since both respond in a similar fashion to visual exposure during early life.

To further explore the role of photoreceptor cell terminal plasticity in visual coding, we stimulated optomotor turning behavior at 100% light intensity but gradually restricted the frontal visual field of the fly. As expected, the more of the frontal visual scenery shown to the fly, the stronger was its behavioral response. Interestingly, optomotor turning responses of DD-flies are already saturated when only 45° of the maximal visual scenery are visible, supporting the above idea that the coding efficiency is enhanced in animals with small photoreceptor terminal volumes (Fig. 2c). Conversely, LL-reared flies display gradual response-increases over the entire visual scenery. This indicates that a reduction of visual coding efficiency allows the transmitted visual signals to be integrated within the optic system, in order to produce a fine graded behavioral response. Thus, the coding capacity of the visual system, computed as the quotient of the optomotor response at 360° stimulation versus that at 30°–60°, is significantly increased in LL-reared flies over DD-reared animals (inset in Fig. 2c).

### Circadian Changes in the Lamina

Although changes in lamina volume and in optomotor sensitivity are correlated, one cannot exclude the possibility that the observed behavioral plasticity has its origin in effects of visual experience on brain centers downstream of the lamina neuropil (e.g. the lobula plate). We therefore looked for other evidence linking morphological changes in lamina to behavioral changes.

There is evidence that in *Drosophila* [9], [10], and in large flies [12], cell populations within the visual neuropils can express circadian rhythms. Plasticity of sensory-system cells might optimize the visual system to operate during times of changing illumination at dawn and dusk, is the times when *Drosophila* are most active. We therefore examined the optic lobes of adult *Drosophila* flies both on EM- and light microscopy level. In addition, we took advantage of the many known circadian rhythm mutations to selectively express proteins coded by clock genes.

We first examined cartridge cross-sections of wild-type light/dark- (LD-) reared flies at 1, 7, 12, 17 and 0 hours Zeitgeber Time (‘Zeitgeber Time’: ZT 0  =  ‘lights-on’; ZT 12  =  ‘lights off’). Axon sizes increased during the day, reaching a peak at midday, then decreased to a minimum at midnight, increasing again (Fig. 3a, c); the circumference of the membrane surrounding the photoreceptor terminals also changed, closely following the circadian pattern of the cross-sectional area. In line with this, the presynaptic T-Bars residing on the membranes showed circadian variations, with a peak during daytime and a trough at night. The abundance of epithelial glial cell extensions also varies with time of day (Fig. 3e): at night, deep capitate projections were twice as numerous as during the day, whereas shallow capitate projections were more numerous during the day than at night. Fitting the data for deep and shallow projections to a sine-wave function revealed a significant (p<0.05) periodicity of 24 hours for both types of glia cell projections, and the respective peaks and troughs of the two curves were out of phase by approximately twelve hours.

Taken together, in both light-dependent plasticity of the lamina (LL versus DD; Fig. 1), as well as in circadian plasticity (Fig. 3), we found photoreceptor terminal area, membrane circumference, residing synapses and epithelial glia cell invaginations to be highly modifiable. The daytime is comparable to the LL-type state, and nighttime resembles the DD-type state. The finding that the plasticity detected at the EM-level leads to morphological changes detectable at the light-microscopy level - and vice versa - allowed us to perform a large scale analysis of lamina volume with a high temporal resolution. The combined data for lamina volume from several independent experiments (overall data from almost 1.700 flies are contained in the corresponding Fig. 4a) revealed robust circadian oscillations with an amplitude of up to 35% and a mean period of 23.7 h under constant light conditions (LL and DD) and under cycling illumination (LD). The rhythm of lamina volume oscillations was independent of visual stimulation over at least the first six days of adulthood (Fig. 4a). The observed volume oscillations are superimposed on mean lamina sizes which have been established during the critical period according to the overall light exposure (LL or DD). Under LD-conditions, the observed volume oscillations are clearly not induced by the day/night and night/day transfers since they anticipated these events. For instance, the lamina volume is down regulated between ZT 9–12 in anticipation of the coming subjective night, and lamina volume increases during ZT 21-0, phenotypically anticipating day. This data suggests that an endogenous circadian mechanism controls the continuous volume plasticity of the photoreceptor synaptic terminals. We did not detect daily volume oscillations in other visual areas such as the medulla, the lobula plate and the calyces of the mushroom bodies (data not shown).

### Parallel Pathways Control Lamina Plasticity

In null-mutants of the core circadian clock genes *period* [*per^01^*; 18], [19] and *timeless* [*tim^01^*; 20], [21] oscillations in lamina volume are abolished in the absence of circadian changes in light levels (open and filled circles in Fig. 4c), but are present in cycling illumination (LD, half-filled symbols Fig. 4c). Closer examination of the volume changes under LD conditions shows significant but slow lamina swelling during light exposure and rapid shrinkage during darkness (black-and-white squares in Fig. 4c). The timing of these structural alterations is, however, not in “anticipation” of day/night or night/day light changes. To further discriminate the light-driven input to the lamina from the circadian we tested flies mutant in the gene *no receptor potential A* (*norpA^P24^*), which are defective in the phototransduction cascade [22]. They displayed a robust rhythm of lamina volumes which was independent of the actual light-rearing conditions (Fig. 4c; best sinusoidal fit for LL: F(1,98) = 3.9; p<0.05; LD: F(1,84) = 4.9, p<0.05; DD: F(1,102) = 7.9, p<0.01). Both, the absolute lamina volumes after LL-, LD- or DD-rearing, and the time course of photoreceptor terminal plasticity (volume max./min. at ZT 6 and ZT 18, respectively; Fig. 4c) were similar in *norpA^P24^* mutants and in dark-reared wild-type flies (Fig. 4a). Altogether the data suggest that circadian fluctuations of PER-protein levels, such as those found in photoreceptor cell nuclei of wild-type and *norpA^P24^*-mutant flies [19], can affect lamina volume and photoreceptor terminals. In addition, since *norpA^P24^* mutant animals can be synchronized with regards to their locomotor activity rhythms [23], daily changes in light exposure can regulate lamina volume, but by a different mechanism from those regulating light-induced plasticity of that structure.

Transgenic expression of PER-protein restricted to photoreceptor cells of *per^01^*-mutants can reconstitute the circadian clock in these cells [24]. In the next experiments we could show that it also rescues the volumetric plasticity of the lamina (Fig. 4d). The rescued volume oscillations of photoreceptor cell terminals in LL and DD conditions (Fig. 4d) displayed dampened amplitude and a time-course delay of a few hours. LD-cycling of the rescued flies appeared to be well matched to the wild type. These phenotypes are consistent with a previous study analysing the molecular rescue of the PER-protein cycle in dark-reared animals of the same genotype [24]. We therefore conclude that a functioning circadian clock in photoreceptor cells R1-6 is sufficient to control photoreceptor terminal plasticity resulting in gross morphological changes of lamina volume.

The genetic dissection of lamina plasticity has shown that both circadian- and light-dependent mechanisms regulate synaptic terminal volumes. In wild-type photoreceptor cells these two mechanisms seem to converge: visual experience caused small, but significant modifications of the time course and amplitude of lamina volume oscillations, being most apparent at ZT 9 (Fig. 4b; (F(2,191) = 5.5, p<0.005, ANOVA). Here, dark-reared animals (DD) showed a down-regulation of the lamina volume, while visually experienced flies (LD) were somewhat delayed in this down-regulation, and constant light (LL) drove the system to maximal amplitude. The two qualitatively different control mechanisms may serve to optimize this sensory system so it can cope with both long-term, unpredictable changes in light levels (e.g. LL vs. DD), and with predictable short-terms changes.

### Behavioral Sensitivity and Photoreceptor Plasticity

If the changes in lamina volume and photoreceptor terminals are part of an optimization of the visual system, they should be accompanied by matching changes in visual-system function. We therefore reared flies in a 12 h light/dark cycle (LD) and analyzed their optomotor behavior throughout a 24 h period. Both, the sensitivity of optomotor responses as well as the dynamic range of visual behavior displayed circadian oscillations with an anti-phasic time course. The optomotor response at low light levels is highest during the night and lowest during day-time hours (F (1,281) = 8,29; p<0.005). Conversely, the dynamic range of the flies' visual performance had a peak at day and a low at night (statistical test between the two peaks: p<0.05). Both curves are anti-phasic by about 12 hours (Fig. 5a, b). These data suggest that the visual system of *Drosophila* is capable of increasing its behaviorally relevant sensitivity at the expense of its dynamic range and vice versa. The time course of these oscillations in behavioral sensitivity is strongly correlated with the one observed for lamina volume plasticity (e.g. Fig. 4a).

In the final experiments we took the large day/night differences in optomotor response as a benchmark to test for day/night and subjective day/night changes, and thus for circadian rhythmicity in optomotor behavior. Consistent with the previous findings, the day/night differences in behavioral sensitivity were found again in LD-reared wild-type flies (Fig. 5a, b) and *per^01^* mutant animals. When dark-reared flies were tested at their subjective day- or night-time hours, differences could still be found in wild type (F(1,24) = 4.9, p<0.05), but they were completely abolished in dark-reared *per^01^* clock mutant flies (compare left to middle panel in Fig. 5c). In the final, critical experiment we tested flies with the expression of PER-protein just in the photoreceptor cells (*per^01^; Rh1(-180)-per-1/+*) once more. We found a lower optomotor behavior performance at subjective day than at subjective night (F(1,40) = 4.8, p<0.05; right panel in Fig. 5c), suggesting that the rescued PER expression in the photoreceptor cells exclusively rescues both volumetric plasticity of the lamina (Fig. 4d) and circadian changes in visual behavior. Thus, lamina volume and sensitivity of optomotor behavior are both regulated by two distinct mechanisms: one circadian involving expression of the PER-protein and a second that is experience dependent and involves the phototransduction cascade in the photoreceptors. We therefore finally conclude that a functioning circadian clock in photoreceptor cells R1-6 is sufficient to control photoreceptor terminal plasticity and thereby to regulate the changes in sensitivity of optomotor behavior.

## Discussion

**Figure 6:**
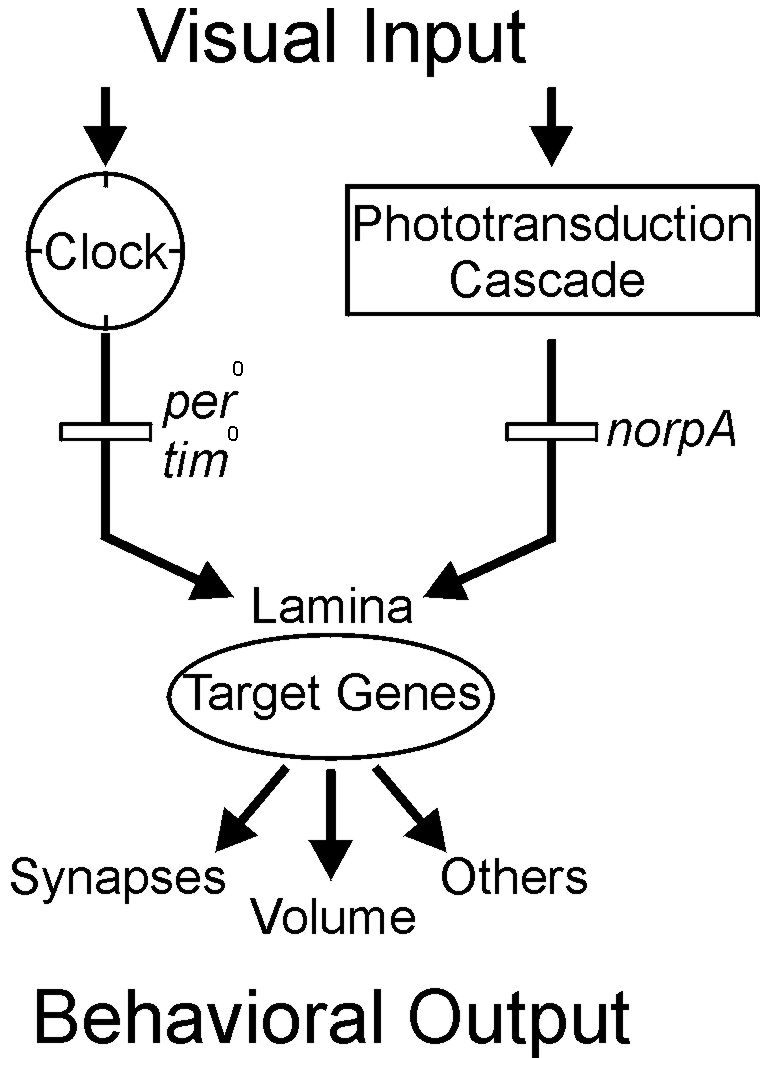
Model of parallel pathways. Light serves at least two functions in the visual system of *Drosophila*, it entrains and keeps the autonomous circadian clock of photoreceptors in phase and it triggers the phototransduction cascade. Both cellular mechanisms are active in parallel in photoreceptor cells and both converge in the volume control of their synaptic terminals in the lamina. The neuronal readout of this peripherally controlled morphological and functional plasticity is further computed downstream of the photoreceptor terminals, within the lamina and/or e.g. in the lobula plate, to instruct the appropriate behavior.

Our experiments show that volume plasticity of photoreceptor synaptic terminals is controlled by the parallel actions of both endogenous circadian- and vision-dependent mechanisms. As our genetic analyses suggest, the circadian input is independent from the phototransduction cascade and is synchronized by light/dark cycles. Visual input strongly depends on a functioning phototransduction cascade. We propose that both input pathways work in parallel and contribute to the period and phasing of the lamina oscillation and the absolute volume of the lamina (Fig. 6). The expression of the clock protein PER exclusively in the photoreceptor cells rescued both structural and behavioral circadian rhythmicity. Therefore, we conclude that the functioning circadian machinery in photoreceptor cells is sufficient to control visual coding efficiency in *Drosophila*. In addition, endothelial glia cells [25] and the large monopolar cells, L1 and L2 [9], [10] might contribute to the output of this circadian network. The specificity of the observed plasticity in the first optic neuropil suggests that this plasticity might participate in the transduction and processing of primary sensory signals rather than in direct photoreception. Our findings do not exclude the possibility, however, that the autonomous network in the periphery of the visual system is affected by circadian oscillators in other parts of the brain such as the axonal terminals of the PDF circuit [11; for large flies: 9], although we have no indication that this is the case.

We are aware that our observation of a sustained circadian rhythm of the lamina under LL-conditions appears to be counter-intuitive in light of the literature on circadian rhythms and their abolishment under LL-conditions [e.g. 18], [21]. Nevertheless, we have demonstrated sufficient data to state that circadian rhythm in this newly described circadian output in *Drosophila* persists under LL-conditions, although we have no molecular explanation for this finding thus far. Alternatively, an external Zeitgeber such as temperature shifts, noise, or changing levels of CO_2_ between day and night could induce the circadian changes in the lamina under LL conditions. However, in control experiments (data not shown) where flies lived under LL-conditions during all stages of their development, measurable circadian rhythms in the lamina were abolished. Interestingly, recent literature [26], [27] provides examples of genetic variants in *Drosophila* which can sustain circadian rhythms under LL-conditions. This observation invites further experiments to shed new light on our present findings.

The peripheral plasticity at the level of first synaptic signal computation governs visual behavior of adult *Drosophila melanogaster*, as demonstrated by the optomotor turning response. A twofold autonomy of behaviorally relevant visual signal processing appears to be well matched to the visual ecology of *Drosophila melanogaster*. Under light-dark conditions, most flies show a bimodal activity pattern with a strong activity-peak in the morning, around 1 lights-on, and an evening peak around lights-off [28]. In line with these behavioral observations, the largest changes in lamina volume were observed for the three-hour intervals preceding lights-on in the morning and lights-off in the evening, overall lamina changes were at a rate of 15% per three hours during that time. These data suggest that the circadian system optimally adapts the visual system of *Drosophila* to the ambient light environment thereby guaranteeing the best vision at dawn and dusk.

Diurnal visual optimization appears to have diverged during evolution. In the majority of systems, including cockroaches [29], large flies [9] horseshoe crab, *Limulus polyphemus* [2], and vertebrates [30], the circadian pacemakers governing circadian rhythms in the visual system have their origin in central brain structures. For example, in *Musca domestica* large PDH-immunoreactive fibre tracts travel from the accessory medulla back to the lamina where they seem to propagate circadian changes to L1 and L2, but leave the volume and synaptic frequencies of photoreceptor cell terminals unaffected. In contrast, in *Drosophila*, independent circadian pacemakers have been found to operate autonomously in many tissues [31], [32], [33], and direct, for example, daily changes in olfactory responses in the antennal neurons [34], [35]. We therefore suggest that the robust rhythmic activity of clock genes in photoreceptor nuclei and epithelial glial cells in the lamina [36], [37] is part of the underlying molecular machinery of another autonomous, self-sustained circadian output system. In *Drosophila melanogster*, the periphery of the visual system can therefore be regarded as a plastic neuronal network in itself, modifiable at different stages of computation, with behavioral flexibility as its emergent property.
